# Precise bonebridge implantation in challenging cases: a novel approach using virtual planning and electromagnetic navigation

**DOI:** 10.1007/s00405-025-09786-y

**Published:** 2025-11-04

**Authors:** Nils Kristian Prenzler, Susan Busch, Niels Rudnik, Thomas Lenarz, Daniel Schurzig

**Affiliations:** 1 Dept. of Otorhinolaryngology, Hannover Medical School, Hannover, Germany; 2 Cluster of Excellence “Hearing4all”, Oldenburg, Germany; 3 MED-EL Research Center, Hannover, Germany

**Keywords:** Bone conduction, Bone-anchored hearing aids, Implant fixation, Safety, Navigated surgery, Bone thickness

## Abstract

**Background:**

The second generation of the Bonebridge (BB) bone conduction implant was shown to be a viable option even for younger children with conductive or mixed hearing loss. However, preoperative imaging often shows only small areas where the FMT (4.5 mm) or screws (4.0 mm) can be fully and safely inserted without the need to use lifts. Navigation systems allow precise placement of the device and prevent potential complications such as dural or vascular injuries. The latest version of the preoperative planning software Otoplan® allows to assess bone thickness and perform virtual implantation.

**Materials & methods:**

Six children between 3 and 12 years of age underwent BB implantation using Otoplan® with export of the planned BB position to a navigation system. Prior to an intraoperative cone beam CT (CBCT), 3 marker screws were placed in the temporal bone. Images were loaded into Otoplan® to virtually define the optimal BB position and export the corresponding model. CBCT scan and model were then loaded into an electromagnetic navigation system. The screws were used to accurately register the system, and the planned BB placement was projected onto the patient. BB implantation was performed accordingly and finally the marker screws were removed. Possible complications were monitored and the audiological success was measured using an age-appropriate speech test.

**Results:**

Bone thickness at the screw location was over 4.0 mm in every case, documenting the accuracy of the procedure. No medical complications occurred intraoperatively, during the immediate hospital stay, or up to and including the initial fitting 4–6 weeks after implantation. Speech test results were greater than or equal to 90% in all measurable patients.

**Conclusion:**

Virtual implantation with Otoplan® can be loaded into a navigation system to mark the safe position of the screws on the temporal bone. Projecting the planned position onto the patient using navigation is a practical tool that can make implantation more reliable and safer for patients.

## Introduction

Bone conduction implants (BCIs) have been available for decades for patients with conductive, sensorineural or mixed hearing loss or unilateral deafness for whom the use of hearing aids is not possible or unsuccessful [7, 15]. Starting with percutaneous solutions, the introduction of several versions and generations of transcutaneous systems [24] demonstrated that these devices appear to be superior in terms of comfort, lower complication rates [9, 10, 18, 20] and cost-effectiveness [3]. As long as there are no contraindications such as bone conduction thresholds exceeding the indication criteria or the medical need for certain c-MRI checks [17, 26], a transcutaneous system should hence be favored.

Children, especially those with auditory canal atresia or other malformations, also benefit from transcutaneous solutions, but insufficient bone thickness can limit implantation options [1, 6, 11, 30]. On the one hand, the screws necessary for implant fixation require a certain bone thickness in order to adequately transfer sound energy to the bone. On the other hand, spatial limitations regarding neighboring anatomical structures such as the sigmoid sinus, emissary veins, dura and auricle must also be taken into account. Injuring these structures can lead to bleeding or CSF leakage, and compression by the implant can lead to complications like sinus vein thrombosis [13] or increased intracranial pressure [1].

Spatial constraints also play a role for adults with unilateral deafness after ipsilateral tumor surgery, which is another common indication for BCIs: after translabyrinthine or retrosigmoid acoustic neuroma excision, for instance, areas of temporal bone relevant for implantation may be missing.

For demanding cases like the ones mentioned above, Otoplan® (CASCINATION AG, Bern, Switzerland in collaboration with MED-EL, Innsbruck, Austria) was developed to determine possible implant positions in preoperative CT scans for the Bonebridge BCI system (MED-EL) [30]. In case of this BCI, two screws fixate the implant 4.0 mm deep in the bone and are crucial for sufficient sound transfer. Additionally, the second-generation FMT (BCI 602) of the Bonebridge is inserted 4.5 mm into the bone, which is particularly important to consider regarding potential sinus or dura compression. Otoplan® can generate heat maps of the skull thickness within the potential implantation area, indicating regions where the bone is thick enough for the screws and/or FMT. It also allows for virtual positioning of the implant prior to surgery to determine the optimal implant location. Unfortunately, up to now it has been difficult to accurately project this location onto the actual patient during surgery, as crucial surgical landmarks are missing especially in the case of anatomical variants. This is particularly disadvantageous in cases where the implantable area on the skull is very narrow, requiring accordingly high accuracy in projecting the planned implant location onto the patient.

In order to address this gap in the workflow, we employed the export functionality of the latest version of Otoplan® to derive a temporal bone model including the planned implant location. This model was imported into a surgical navigation system to precisely determine the optimal implant location on the actual patient during surgery. This paper details this procedure and presents results from a consecutive series of cases where safe implantation was achieved despite low bone thickness.

## Materials and methods

### Patient cohort

In this retrospective case series, all patients who underwent Bonebridge implantation with the help of navigation at our tertiary university hospital were included. Between 2022 and 2024, six children, including 4 boys and 2 girls could be identified. The age at implantation ranged between 3.5 and 12.9 years. The most common cause of hearing impairment was ear canal atresia (3 cases unilateral, 1 case bilateral). One child suffered from chronic otitis media and Eustachian tube dysfunction, which was associated with a combined conductive and sensorineural hearing loss. One girl was treated with a BB as a CROS system due to ipsilateral auditory nerve aplasia. All children had previously tried a Baha or Ponto on headband.

### Audiological assessment

As part of the routine diagnostic procedure directly before surgery, BERA (Brainstem Evoked Response Audiometry) tests for air and bone conduction (masked) were performed on all children, and the corresponding thresholds were determined. If possible, preoperative pure-tone audiometry was conducted as well. However, the thresholds from pure-tone audiometry were only deemed reliable in one child, so BERA thresholds were used for indication in the other patients. Because of the severe degree of hearing loss, unaided speech testing was not conducted preoperatively in these children.

### Bonebridge implantation

All surgeries were performed under general anesthesia. After additional infiltration anesthesia, a retroauricular incision was made, and a skin flap was created, preserving the temporalis muscle which was incised posteriorly along the linea temporalis. A periosteal (Fisch-) flap was then formed, which remained pedicled anteriorly at the ear canal. The temporalis muscle was lifted upwards, exposing the mastoid plane. Three self-cutting MICRO screws (1.5 × 3.5 mm, Zimmer Biomet, Warsaw, USA), placed as far apart as possible within the exposed skull surface, were inserted for navigation, and the surgical access was temporarily closed.

A CBCT (Cone Beam Computed Tomography) scan of the temporal bone was performed intraoperatively. The dataset was imported into Otoplan®, and the visualized bone thickness was set to 4.0 mm (heat map). A virtual implantation of the BB was then carried out, determining a position that would allow for safe placement of the BB and its fixation screws in all three planes without needing lifts, and without the screws exiting the skull bone medially toward the dura. The navigation dataset was simultaneously loaded into an electromagnetic navigation system (SCOPIS Hybrid Navigation System, Stryker GmbH & Co. KG, Duisburg, Germany).

Subsequently, the surgical access was reopened, and the navigation system was registered using landmarks (the 3 navigation screws) and an extended surface (the surface of the skull bone). After successful registration (with an error below 0.3 mm), the implant position modeled in Otoplan® was imported into the initial navigation dataset.

Next, the simulated/modeled BB screw positions were located on the skull surface of the patient using the navigation system’s pointer. The positions were marked with a pen, and the target location of the bone bed was subsequently outlined between the screws using the BB template supplied with each implant set.

Once the bed was prepared and the template could fully sink into the bone, it was visually checked if the bone at the edge of the bed closest to the intended screw locations appeared to be sufficiently thick (i.e., 4 mm or more) to safely fixate the implant with the screws without damaging the dura or other structures.

Finally, the implant was inserted, and the FMT was fixed using the self-cutting screws provided by the manufacturer.

Lastly, the three navigation screws were removed, and a multi-layered wound closure was performed. The duration of the surgery and any complications (including postoperative) were documented.

## Results

### Audiological assessment

**Fig. 1:**
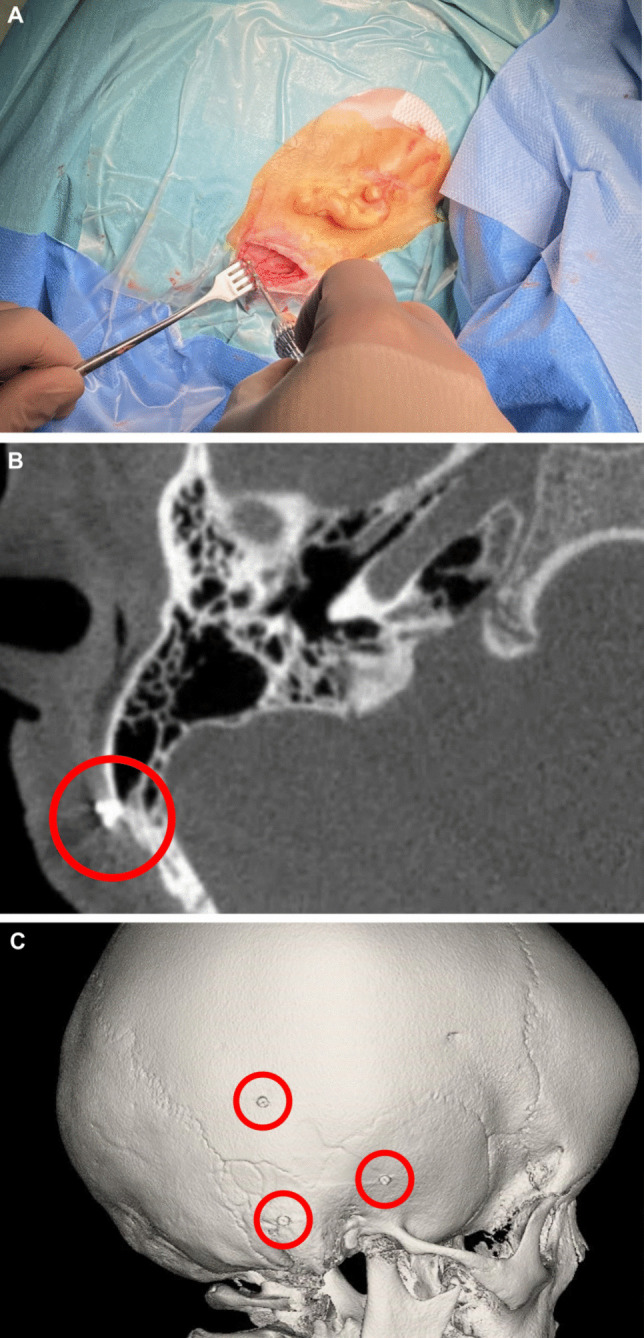
Implantation of screws for electromagnetic navigation **A**) Surgical incision and implantation of self-cutting screws. **B**) Axial plane from an intraoperative CBCT scan showing an implanted screw. **C**) 3D reconstruction from the CBCT scan displaying the locations of all three screws

**Fig. 2:**
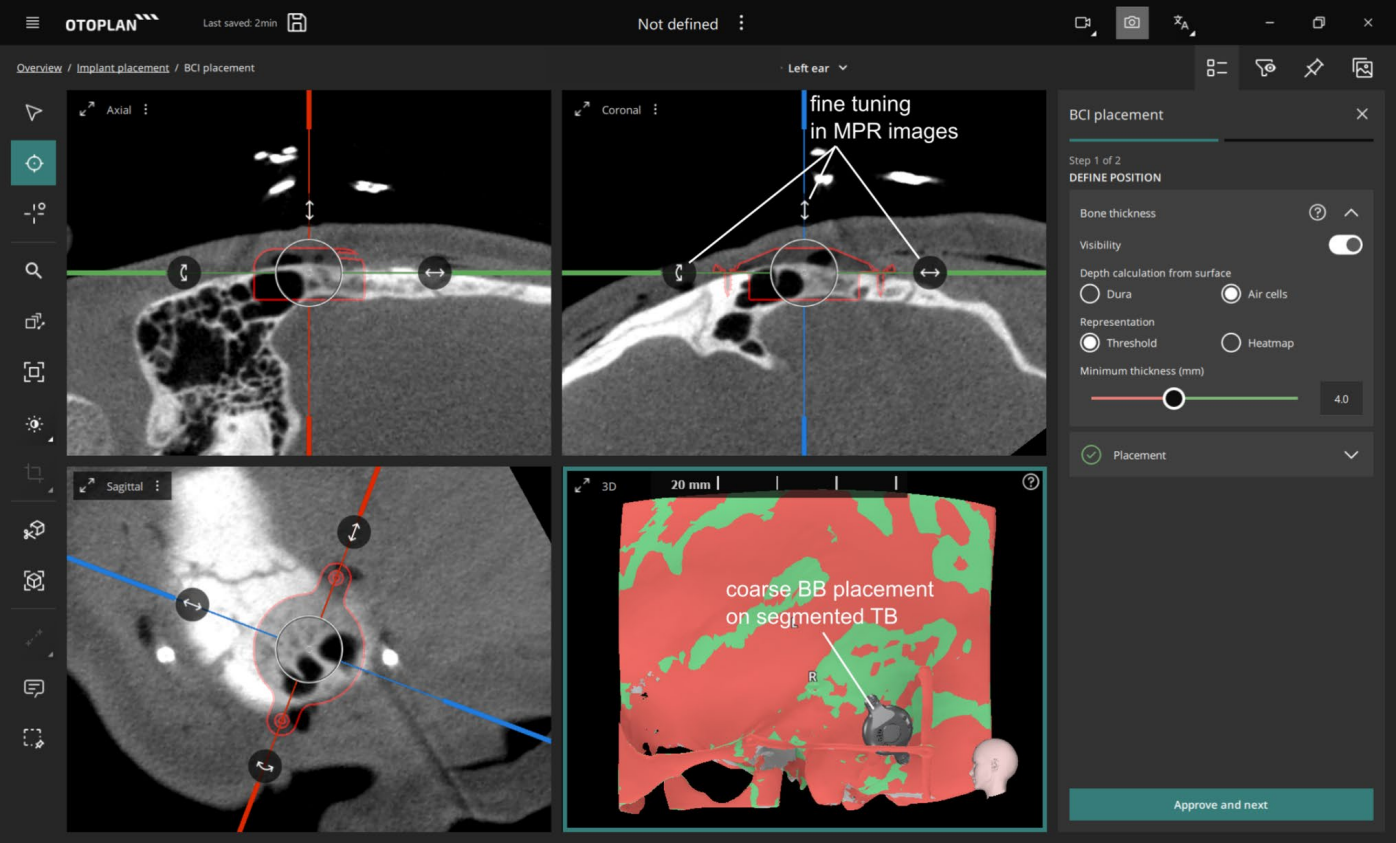
Screenshot of intraoperative planning for BCI and screw placement. The planning interface demonstrates a reasonable position for the Bone Conduction Implant (BCI) and its screws: Top right: The cortical thickness is set to 4.0 mm. In the heatmap displayed in the bottom, all green areas indicate sufficient bone thickness for the fixation screws required for the Bonebridge. The Bonebridge can be virtually placed, and its position fine-tuned in three planes for optimal alignment. Particularly, the coronal plane is showing the fixation screws relative to the bone thickness at the intended screw locations

**Fig. 3:**
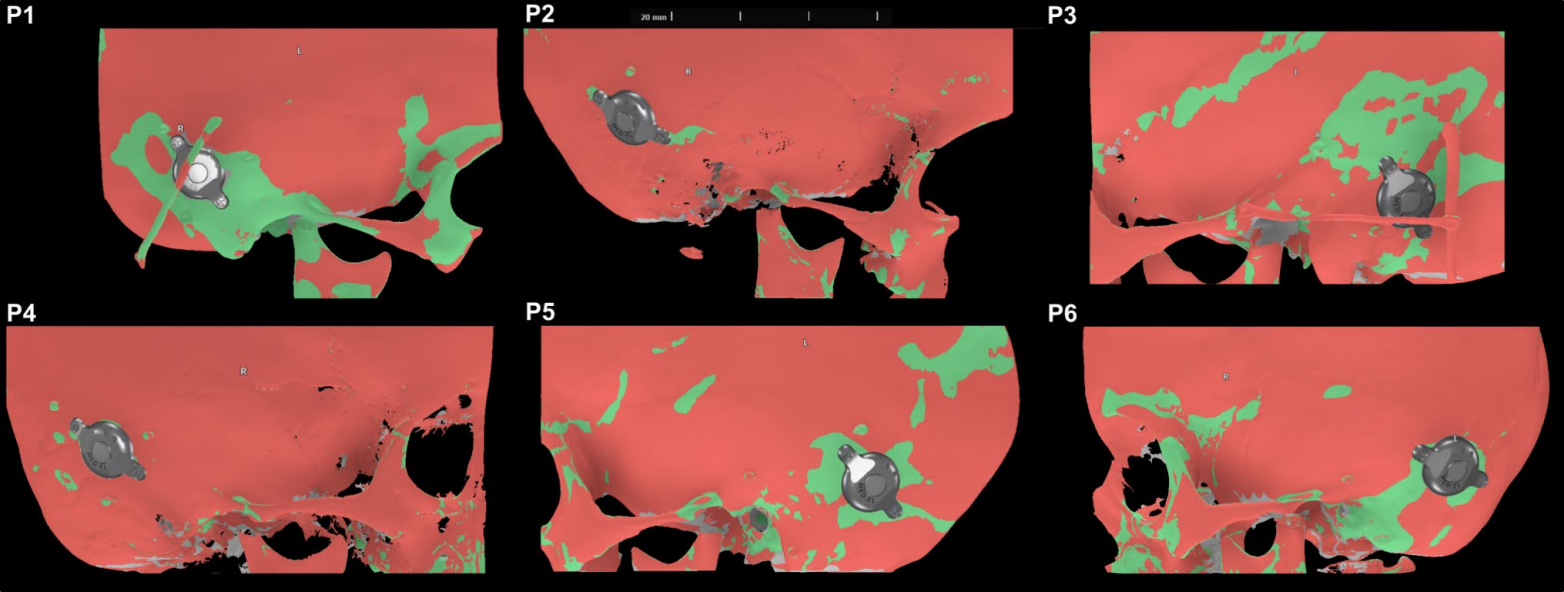
Heatmaps with virtually implanted bonebridge across all 6 patients. The heatmaps illustrate the bone thickness distribution for all six patients: Green areas represent regions with a bone thickness of at least 4.0 mm, suitable for screw placement of the Bonebridge. The virtual placement of the Bonebridge is shown for each patient, ensuring optimal positioning within areas of adequate bone thickness

Postoperatively, the hearing outcome with the BB of the first five children was measured using age-appropriate speech tests (Mainz and Göttingen children´s test, Freiburg monosyllable test) in quiet at 65 dB SPL. Test results from the routine fitting appointment 2.0 ± 0.2 months after first fitting (7.0 months after first fit for patient 3) were used for Table 1. For Patient 6, no suitable native language speech tests were available, but the parents reported high satisfaction with the subjective benefit of their child (Figs. 1, 2 and 3).

**Table 1:** Overview of patients with navigation-assisted implantation

Number	Sex	Side	Age at Implantation (years)	BCpre PTA4 (dB nHL)	ACpre PTA4 (dB nHL)	Etiology	Duration of surgery	Speech-test at 65 dB (%)
1	Male	R	3.5	30*	70*	Ear canal atresia	3h54min §	90 M
2	Male	R	4.0	30*	80*	Ear canal atresia	2h45min	90 M
3	Male	L	12.9	5	65	Ear canal atresia	1h58min	90 *F*
4	Male	R	4.4	20*	70*	Ear canal atresia	2h04min	100 *G*
5	Female	L	4.6	n.d	20*$	Auditory nerve aplasia (SSD)	2h00min	90 *F*
6	Female	L	5.3	40*	80*	Chronic otitis media	2h07min	n.a. #

PTA4: Average of thresholds at 0.5, 1, 2, and 4 kHz; *: BERA thresholds; § Bilateral surgery, *n.d.* not done, *n.a.* not applicable, $ SSD patient, threshold from contralateral side, *M* Mainz children´s test, *F* Freiburg monosyllables test, *G* Göttingen children´s test

^#^: No suitable speech tests were available for the native language of this patient

### Bonebridge implantation

**Fig. 4:**
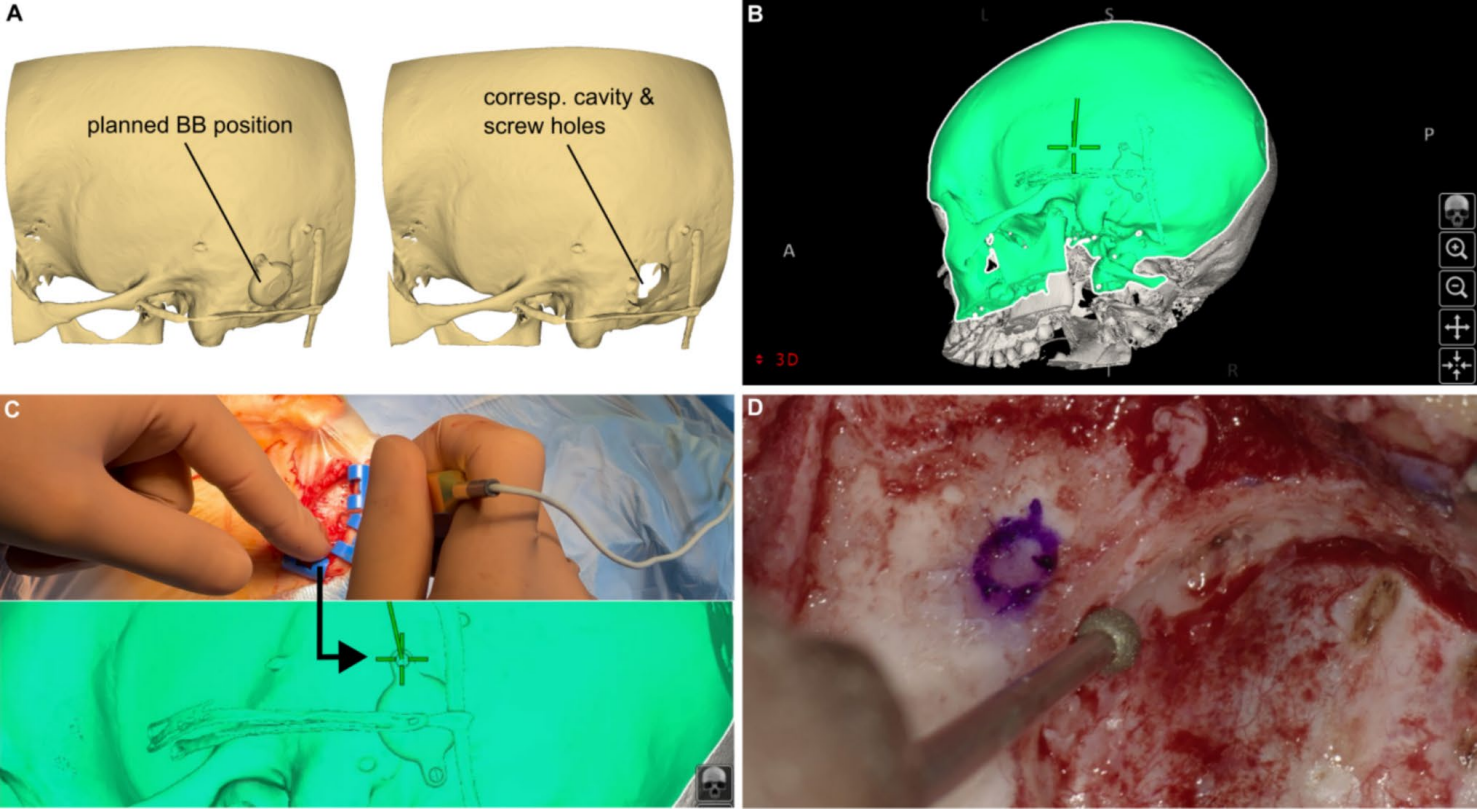
Workflow of intraoperative navigation. **A**) Export of the virtual implantation plan from OTOPLAN®. **B**) Import of the virtual implantation into the navigation system and overlaying it with the intraoperative CBCT dataset and the OTOPLAN® model. **C**) Localization of the planned positions for the two screws using a sterile pointer. **D**) Visual verification of sufficient bone thickness at the implant bed

All surgeries were performed without complications under uneventful intubation anesthesia. The average operation time from the first incision for implanting the screws to suturing after Bonebridge implantation was 148 ± 41 min (Table 1). In all cases, the dura and the sigmoid or transverse sinus were exposed. Sufficient bone thickness was visually confirmed by checking the bone thickness directly adjacent to the two marked screw positions (Fig. 4D) in all cases. In one case of bilateral navigation, reliable registration with the electromagnetic navigation system was not possible on the second side, which we attributed to interference with the field generator caused by the already implanted FMT on the opposite side.

No medical complications occurred intraoperatively, during the immediate hospital stay, or up to and including the initial fitting 4–6 weeks after implantation.

## Discussion

A variety of studies have documented the audiological effectiveness and safety of various transcutaneous bone conduction systems, including the BB by MED-EL [2, 21, 24]. Implantation in challenging anatomies, such as in children from the age of 3, has also been successfully carried out [2] age:5–17 years [19], age:10–17 years [6], age:8–18 years [23], age:6–16 years [30], age:3–11 years [4], age: from 5 years on). However, it is crucial to mention the necessity of adequate bone thickness for a safe implantation.

Bone conduction systems are highly flexible and their placement can be easily adapted to the individual situation in most patients. Experimental studies suggest that positioning the implant near the outer ear canal or close to the labyrinthine structures is audiologically effective [8, 27, 28]. However, the optimal position recommended by each manufacturer may not be feasible due to bone thickness, such as in small children or patients after temporal bone tumor resection. Furthermore, the quality of the surrounding bone can influence the transmission quality of the bone conduction implants [29]. In addition to anatomical limitations, functional "sweet spots" may also exist, which can be determined from clinical imaging and should be considered during surgery. These circumstances motivated the development of the present procedure, which, for the first time, allows for true precision in optimal individual positioning of the BB.

The surgical procedure presented here allowed for the safe implantation of the BB, even with only very small islands of sufficient bone thickness (Fig. 3). The bone thickness was adequate for screw placement and minimized the impression on the dura or sigmoid sinus beneath the FMT. The focus was on a secure and functional placement of the screws rather than potential impressions on the dura. With the new version of the BB 602, which only penetrates 4.5 mm below the bone surface, this implant bed is comparable to the bone bed created during cochlear implantation to house the anterior portion of the antenna. In this procedure, widely regarded as safe, a bony island is created, also involving an impression of the dura to a similar extent [14].

This study is the first one to describe image-guided, precisely planned implantation of the BB in patients using Otoplan´s thickness mapping and novel model export function.

In a study by Kong et al. [12], navigation was used intraoperatively to identify a preoperatively planned position based on 3D modeling, albeit in a single case. The authors utilized BB fast view (CEIT, Guipuzcoa, Spain), a 3D simulation software, and performed registration using fiducial markers and surface matching in a patient with normal mastoid anatomy and a normally developed pinna.

The technique demonstrated here differs essentially in two key aspects. As shown in the pediatric cases presented, identifying areas with sufficient bone thickness plays a crucial role. Otoplan® offers a heatmap visualization that facilitates this process and was essential in these cases to determine an implant position that allowed for a lift-free placement (see Fig. 3).

Furthermore, in the study by Kong et al., surface registration was used. However, especially in the cases with aural atresia presented here, this method would have been too imprecise. Therefore, the workflow described in our study relied on the use of screws for registration.

To locate a planned BCI position in situ, landmarks such as the zygomatic arch, mastoid tip, and lateral orbital rim can be used. The correct intraoperative positioning of the implant and epithesis anchors was, for example, determined by Seiwert et al. using the intersection points of the respective distances of defined landmarks [22]. However, determining the exact designated position is challenging, and in cases of malformations or postoperative conditions, landmarks may be entirely absent.

Another option is the use of 3D printed masks, which can be precisely fitted to the surface in situ. These masks can indicate the planned screw or BCI position through an opening in the material. Canzi et al. published a temporal bone study where they found a mean error of 0.13 mm when comparing the transducer site of a 3D printed model to that of the corresponding cadaveric human temporal bone [5]. Another group used Surface Template-Assisted Marker Positioning (STAMP). In this approach, a small mask is 3D-printed to fit precisely onto the surface of the temporal bone or skull. The mask is designed to sit securely, with holes in the material indicating the positions for the BCI and screws. This method was utilized in three surgeries, exclusively performed in adult patients. However, the required surgical access for this technique might be too extensive to be used in children. Additionally, the availability of a 3D printer is a prerequisite for employing this method [16].

The duration of the BB implantation surgery according to the present approach is longer than the usual duration for Bonebridge implantation: the average duration of surgery was 57 ± 19 min for adults and 57 ± 20 min for pediatric patients in a multicenter study on the 602 version of the Bonebridge [25]. The present duration can likely be reduced in the future with more routine, especially in the processing of DICOM data and its fusion with the navigation system. It should also be noted that this case series was exclusively conducted in children. In adults, it is possible to perform the incision and screw placement under local anesthesia, scan the patient while awake, and have the model and fusion with the navigation system already prepared for the actual implantation. However, skull bone thickness is typically globally sufficient for screw fixation amongst adult patients, making the presented navigation procedure unnecessary. In order to minimize the anesthesia exposure for children, this case series demonstrated that the process could be performed under one single anesthesia, with the total time being roughly comparable to cochlear implantation or reconstructive ear surgery.

Another key finding of the study was that in the bilateral case, the electromagnetic transducer of the first implant interfered with the electromagnetic navigation system in such a way that correct navigation for the second side was not possible. Optical systems should hence be considered as a solution to this issue.

A definitive limitation of this case series is the absence of an objective test to document the intended precision. To address this, a postoperative CBCT examination would be necessary to correlate the planned implant position with the actual achieved position. However, this is simply not feasible due to radiation protection regulations. Even if such imaging were available in these cases, a comparison with a control group implanted without navigation would still not be possible, as postoperative imaging is not part of the clinical routine for bone conduction implants.

The key success factor of this procedure is, rather, that we were able to implant children who, due to insufficient bone thickness, would not have been considered candidates for implantation using conventional DICOM viewers. However, the visualization of small potential bony islands in Otoplan® and their precise implementation enabled safe surgical planning even in these borderline cases, resulting—as demonstrated—in successful and complication-free implantation.

## Conclusion

In summary, this study presents a new workflow applied in six pediatric cases using the latest Otoplan® functionalities. Although this approach is time-consuming and therefore more costly, it enables cochlear implantation even in cases with limited bone availability or challenging anatomy. While the presented heat maps highlighted zones with sufficient bone thickness, future versions should potentially include additional information such as local bone density or distance to the cochlea or outer ear canal, which were shown to be audiologically relevant. These enhancements would make the presented approach interesting for adult patients with sufficient bone thickness as well, allowing for optimization of implantation outcomes in every case.
